# Diabetes Knowledge, Attitudes, and Practices in adults with type 2 diabetes at primary health care clinics in Kimberley South Africa

**DOI:** 10.4102/safp.v66i1.5838

**Published:** 2024-01-29

**Authors:** Moses Alenbalu, Chika K. Egenasi, Wilhelm J. Steinberg, Omololu Aluko

**Affiliations:** 1 Department of Family Medicine, Faculty of Health Sciences, University of the Free State, Bloemfontein, South Africa; 2 Department of Biostatistics, Faculty of Family Medicine, University of the Free State, Bloemfontein, South Africa

**Keywords:** diabetes mellitus, knowledge, attitude, practice, Kimberley, South Africa

## Abstract

**Background:**

Diabetes mellitus (DM) is a common non-communicable disease associated with significant morbidity and mortality globally. It poses a huge public health and economic challenge. People with diabetes need to have adequate knowledge, attitudes and practice (KAP) to prevent complications from diabetes. This study aims to evaluate the KAP towards diabetes among type 2 diabetes mellitus (T2DM) patients attending primary healthcare clinics in Kimberley.

**Methods:**

A cross-sectional analytical, quantitative questionnaire-based study was done using a convenient sampling method in Sol Plaatje Municipality, Kimberley, Northern Cape.

**Results:**

A total of 363 type 2 diabetic patients took part in the study. Most of the participants (62.0%) were females. Most had good knowledge (67.5%), while 64.5% of the participants showed good attitudes towards diabetes. However, only 35.8% of the participants had good practices towards diabetes. There was a significant association between the participant’s level of education and (1) knowledge and (2) practice, with *p*-values of 0.002 and 0.0075, respectively. No significant association was found between the participant’s level of education and attitudes towards diabetes (*p* = 0.2416).

**Conclusion:**

This study demonstrated good diabetes-related knowledge and attitudes but inadequate practices among participants. Educational programmes to assist patients with diabetes to improve their practice towards diabetes should be encouraged and implemented.

**Contribution:**

This study will help to create awareness of the need for people with diabetes to improve their practices towards diabetes.

## Introduction

Diabetes mellitus (DM) is a clinical syndrome with diverse aetiologies associated with persistently elevated blood glucose and disruptions of carbohydrate, fat and protein metabolism due to defective insulin secretion by the B cells of the pancreas or resistance to insulin use by the body.^1,2,3^ It is a disease that is common across the globe, and it affects people of all races and ethnic groups worldwide.^1,2,4^

The coronavirus disease 2019 (COVID-19) pandemic has provided further insights into why diabetes should be of tremendous public health concern. It was reported that people with diabetes have an increased risk of hospitalisation and mortality from COVID-19 infection.^5,6^

The World Health Organization (WHO) recently reported that the world is in the midst of a diabetes pandemic, with people in Southeast Asia and the Western Pacific most at risk.^7^ An epidemiological study of the burden of diabetes revealed that in 2021, about 537 million persons were living with the disease globally. This was a rapid increase from 2019, when the International Diabetes Federation (IDF) reported that approximately 463 million people had diabetes worldwide.^1,7,8^

In South Africa, the prevalence of diabetes has surged.^9^ This increase has been strongly linked to the increase in the socioeconomic status resulting into a changing consumption habits of most South Africans in the past two decades, leading to dietary changes from the consumption of local organic foods to more processed foods.^4,9,10,11^ In the year 2021, South Africa had the highest diabetes prevalence in Africa, with a prevalence of 11.3%, affecting 4.2 million people aged 20–79 years according to IDF reports. This is significantly increased from the 1.9 million individuals with diabetes in the same age group affected in 2011.^8^ Statistics SA in 2016 reported that the Northern Cape had 115 227 people with diabetes.^12^

Diabetes is significantly associated with a high level of mortality. It accounted for approximately 366 200 deaths in 2019 and 416 000 deaths in 2021 in Africa.^8,11^ The ongoing global increase in diabetes and complications has been widely associated with non-compliance to treatments, resulting in poor glycaemic control.^8^

Healthcare expenditure on diabetes is relatively small in Africa compared to global spending.^13^ In Africa, a total of $3.4 billion was the estimated annual spending on diabetes by all African countries in 2015.^1^ This represents the smallest spending on diabetes by any continent in the world. A pioneering study by Erzse et al. in 2018, which estimated the cost of managing type 2 diabetes mellitus (T2DM) patients in South Africa, reported that more than $198m were spent on diabetes, both on treating the disease and its complications.^14^

Khan et al., in a cross-sectional study in 2019, reported that having poor knowledge about DM and low socioeconomic status play a vital role in understanding the complications.^15^ They suggested that massive efforts should be geared towards public enlightenment to improve diabetes knowledge among rural dwellers in India to prevent most of the complications associated with diabetes.^15,16^ In Indonesia, a study to assess T2DM knowledge, attitudes and practice (KAP) regarding compliance found that the level of education played a pivotal role in the patient’s compliance. However, the interaction between healthcare workers and patients’ understanding of given instructions is also helpful in ensuring patient compliance. It is recommended that more resources be provided to educate T2DM patients to enhance their knowledge and understanding as it is directly linked to developing good attitudes and practices.^17^ Hoque et al., in a study in Durban, South Africa, found a moderate correlation between knowledge of T2DM and positive practices.^18^ They concluded that patients with better knowledge of diabetes would subsequently have positive practices towards diabetes.^18^

Numerous studies have accurately described the importance of KAP about DM and improved glycaemic control and self-care in curbing diabetes-related complications.^14,15,16,17,18^ An unpublished record of the Emergency Centre in Robert Mangaliso Sobukwe Hospital (RMSH) in Kimberley revealed a total of 360 diabetes emergencies between January and December 2019. Therefore, it is essential to ensure that patients have adequate knowledge, positive attitudes and good practices towards the disease to reduce the chances of developing avoidable complications.^19^

It has been reported that being knowledgeable, having a positive attitude and following acceptable practices result in successfully managing T2DM.^4,9^ It is also noteworthy that inadequate knowledge, unhealthy attitudes and poor practices related to T2DM further exacerbate the complications.^4,18^

Although a few studies in South Africa reported gaps in diabetes KAP, none have been conducted in Kimberley.^4,9,14,18^ It is, therefore, pertinent to know if there are gaps in KAP towards diabetes among T2DM patients attending Kimberley’s Primary Health Care (PHC) clinics.

This study aims to evaluate the level of KAP towards diabetes among T2DM patients attending the PHC clinics in Kimberley.

## Method

### Study design

A cross-sectional, analytical, quantitative questionnaire-based study was done.

### Setting

This study was carried out in Sol Plaatje Municipality, in Kimberley, Northern Cape.^20,21^

Northern Cape Province is the biggest Province in South Africa, but with the smallest population of about 1 193 780 as of 2016.^21^ Kimberley, the capital city of the Northern Cape, has a population of 225 041.^21^ There are 12 clinics in the Sol Plaatje Municipality.^21^ Three of these PHC clinics were used for this study: Beaconsfield Clinic, City Clinic and Betty Galeshewe Clinic are all located within the city of Kimberley in Sol Plaatje Municipality. Racial distribution is as follows: black African 63.1%, mixed-race 26.8%, Caucasians 8.0%, Indian or Asian 1.2% and others 0.9%.^21^

### Study population

The study population consisted of adults with T2DM in Sol Plaatje Municipality currently attending Beaconsfield, City or Betty Gaetsewe clinics in Kimberley, Northern Cape from February 2021 to April 2021.

These clinics care for an average of 240 T2DM patients monthly. Beaconsfield Clinic, City Clinic and Betty Gaetsewe clinics saw 2144, 2241 and 2432 T2DM patients in 2020, respectively. These clinics refer their complicated patients, such as those with target organ damage, uncontrolled diabetes and diabetes emergencies, to RMSH for further management.

### Inclusion criteria

Participants were all adults, including pregnant women diagnosed with T2DM by a medical practitioner, 18 years and above, attending three PHC clinics (Beaconsfield, City and Betty Gaetsewe) in Sol Plaatje Municipality, Kimberley. Participants must be willing to provide informed consent.

### Exclusion criteria

Patients < 18 years old, all patients with type 1 diabetes, type 2 diabetes patients presenting with medical emergencies and patients who cannot consent to the study.

### Sampling method and sample size

Convenience sampling was used in this study; patients with T2DM who met the inclusion criteria and who were available and willing were chosen for the data collection. The sample size was estimated from each clinic’s total number of patients with diabetes using Openepi, a software for sample estimation. The calculated sample size was 364 at a 95% confidence interval and 5% of margin error.

### Data collection

Data were collected between February 2021 and April 2021 using a structured, self-administered, confidential questionnaire made available in the city’s three most prominently spoken languages: English, Afrikaans and Setswana. A trained translator translated the English version into the other two languages verbatim. The questionnaire was designed based on a pre-validated questionnaire by Khan et al. in a diabetes KAP study.^15^ Permission to use the questionnaire was obtained via email and adapted to suit the local environment. The questionnaire was further validated by specialists from the Department of Family Medicine at RMSH who are knowledgeable about diabetes.

The questionnaire was categorised into various sections: section 1 consisted of information on the sociodemographic distribution of variables of participants such as age, sex, ethnic group, level of education and employment status. Section 2 covered 20 questions on knowledge of T2DM, such as ‘what is diabetes?’, ‘what are the symptoms of diabetes?’, ‘what are the required lifestyle modifications?’ and ‘what are the complications of diabetes?’. Section 3 covered 9 questions on attitudes towards T2DM. Section 4 covered 10 questions regarding participants’ practices towards T2DM. Scores were calculated as the sum of the correct answers and grouped into ‘Good, Average or Poor’. Knowledge was graded as follows: ‘Good’ knowledge 15–20, ‘Average’ knowledge 10–14, and ‘Poor’ knowledge 0–9 points. Attitudes scores between 7 and 9 points were graded as ‘Good’ attitude, 4–6 points were graded as ‘Average’ attitude, and ‘Poor’ attitude was 0–3 points. Good practice was graded as scores between 7 and 10 points, ‘Average’ practice was graded as scores of 4–6 points, and ‘Poor’ practice was graded as scores between 0 and 3 points.

The first author and a trained research assistant were responsible for collecting data in the three clinics within Sol Plaatje Municipality in Kimberley during working hours, which are 08:00 to 16:00, Monday to Friday, except during public holidays.

Adequate arrangements were made to ensure social distancing, sanitisation and adherence to COVID-19 protocols to avoid spreading the COVID-19 infection during data collection. After seeing the doctors, each participant was briefed about the study. An information sheet was made available for the participants, and they were required to complete a consent form if they agreed to participate in the study. The participant’s right to withdraw or refuse consent was duly explained to each participant. The questionnaires were then administered to each participant capable of completing them. Participants who could not complete the questionnaire because of their inability to read the questions were assisted by the research assistant in a separate room where other participants were not present to maintain confidentiality.

### Pilot study

The questionnaire was piloted using nine patients with T2DM who met the inclusion criteria recruited from Beaconsfield, City and Betty Gaetsewe clinics. This helped to test the suitability of the questionnaire. The biostatistics department of the University of the Free State analysed the data. Data from the pilot study were included as part of the main study, as no adjustment of the questionnaire was needed.

### Data analysis

The collected data were computed on an Excel spreadsheet and analysed using SAS 9.4 software. Descriptive statistics were calculated, namely cumulative frequencies and percentages for categorical data and median. The results are presented in tables and graphs, and the association between variables was determined using appropriate statistical interpretations. Chi-square was used to determine the association between participants’ level of education and KAP. The probability value (*p*-value) of less than 0.05 was accepted as statistically significant.

### Ethical considerations

The protocol for the study was approved by the Health Sciences Research Ethics committee (HSREC) of the University of the Free State with reference number UFS-HSD2021/1633/2501. Approval for data collection was also obtained from the Northern Cape Health Department.

Number coding was used to ensure the confidentiality of the participants’ responses. No names or personal identifiers appeared on any research-related information or datasheet sent for statistical analysis. All paper-based records were kept in a secure location by the researcher and were only accessible to those involved in the study. All information was managed in a strictly professional and confidential manner.

## Results

### Patient/participant profile

A total of 364 participants with T2DM took part in the study, and one questionnaire was rejected due to incomplete answers. Most participants were between the ages of 36 and 55 years (137; 37.7%), closely followed by participants in the age ranges 56–65 years (133; 36.6%), > 65 years (67; 18.5%) and 18–35 years (26; 7.2%).

The majority of those who participated in this study were females, 225 (62.0%), and male participants were 138 (38.0%). Almost half of the participants were married (48.5%); just over one-third were single (38%), separated (5.8%) and divorced (7.7%). Most participants (49%) had up to grade 12 education, while 14.1% had no formal education. Participants with a tertiary and primary level of education accounted for 14.9% and 22.0%, respectively. One-third of the participants (33.3%) were pensioners, whereas unemployed participants were 88 (24.2%). This was nearly equalled by those employed in the private sector (24.5%). Those participants employed by the government accounted for 13.5%.

The participants were predominantly black people (61.2%), while mixed-race and white people accounted for 24.2% and 9.1%, respectively. Indian people, others and people of Asian ethnic descent were 3.0%, 1.7% and 0.8%, respectively.

### Knowledge relating to type 2 diabetes mellitus

Table 1 represents the findings relating to participants’ knowledge of T2DM. Most participants (83.8%) agreed that diabetes is when the blood sugar level is higher than normal. There was also overwhelming agreement among the participants (81.8%), who agreed that excessive urination is a symptom of diabetes. However, only 52.1% believed that increased appetite could be a symptom of diabetes, whereas 26.2% disagreed that increased appetite is a symptom of diabetes. Most of the participants said ‘yes’ and that feeling excessively thirsty and having poor vision could be symptoms of diabetes at 82.9% and 86.8%, respectively.

**TABLE 1:** Participant’s knowledge relating to type 2 diabetes mellitus (*n* = 363).

Questions	Yes		No		Don’t know	
	*n*	%	*n*	%	*n*	%
**What is diabetes?**						
Diabetes is when the blood sugar level is higher than normal?	304	83.8	11	3.0	35	9.6
**Symptoms of diabetes?**						
Excessive urination at night is a symptom of diabetes?	297	81.8	31	8.5	48	13.2
Increased appetite is a symptom of diabetes?	189	52.1	95	26.2	35	9.6
Feeling excessively thirsty is a symptom of diabetes?	301	82.9	37	10.2	25	6.9
Extreme tiredness may be a symptom of diabetes?	229	63.1	80	22.0	54	14.9
Having problem with clear visions could be a symptom of diabetes?	315	86.8	32	8.8	16	4.4
Weight loss may be a symptom of diabetes?	220	60.6	82	22.6	61	16.8
Poor control of diabetes could lead to complications of diabetes?	276	76.0	19	5.2	68	18.7
Diabetes medications are to be taken for life?	317	87.3	18	4.9	28	7.7
How long have you had diabetes?	1–5 years 123	1–5 years 33.9	< 1 year 45	< 1 year 12.4	> 5 years 195	> 5 years 53.7
**This section is aimed to test your understanding of lifestyle modifications**						
People with diabetes should stop smoking cigarettes?	280	77.1	41	11.3	11.6	11.6
People with diabetes should stop drinking alcohol?	290	79.9	38	10.5	9.6	9.6
People with diabetes should stop eating fatty/oily food?	342	94.2	13	3.6	2.2	2.2
People with diabetes should engage in regular exercise?	340	93.7	6	1.7	4.7	4.7
People with diabetes should stop eating food containing sugar?	353	97.3	5	1.4	1.4	1.4
**What are the complications of diabetes?**						
Eyes damage?	348	95.9	6	1.7	9	2.5
Heart problems?	228	62.8	71	19.6	64	17.6
Kidney problems?	273	75.2	50	13.8	40	11.0
Foot ulcer?	290	79.9	24	6.6	49	13.5
Stroke?	251	69.15	35	9.6	77	21.2

Approximately 63% of the respondents believed that extreme tiredness might be a symptom of diabetes. Many participants (60.6%) agreed that weight loss is a symptom of diabetes. In comparison, 76% of the participants believed that if diabetes is poorly controlled, it could result in complications of diabetes. Almost 90% (87.3%) said ‘yes’ that diabetes medications are lifelong treatments. About half of the respondents (53.7%) have had diabetes for over 5 years, while 12.4% have been diabetic for less than a year.

Regarding lifestyle modification, most participants agreed that certain lifestyles needed to be modified by people with diabetes. Most participants, 280 (77.0%) and 290 (79.9%), agreed that people with diabetes should stop using cigarettes and alcohol. Overwhelmingly, 342 (94.0%) respondents agreed that people with diabetes should stop eating fatty and oily food. Approximately 94% know that people with diabetes should engage in regular exercise. Participants (97.3%) agreed that people with diabetes should stop eating food containing sugar.

Many of the participants agreed that diabetes could cause eye (348; 95.9%) and renal (27; 75.2%) complications. At the same time, 62% agreed that cardiovascular conditions could complicate diabetes. There were 77 (21.2%) participants who did not know that cerebrovascular accident (stroke) could be a complication of diabetes, while 251 (69.0%) agreed that stroke is a complication of diabetes. Furthermore, most participants (79.9%) believed foot ulcers could complicate diabetes.

### Attitudes relating to type 2 diabetes mellitus

Table 2 represents the findings regarding attitudes, as well as the total number of respondents and percentage in each question.

**TABLE 2:** Participant’s attitudes relating to type 2 diabetes mellitus (*n* = 363).

Questions	Yes		No		Don’t know	
	*n*	%	*n*	%	*n*	%
Keeping normal blood sugar helps to prevent complications of diabetes?	298	82.1	15	4.1	50	13.8
Diabetes is a serious disease?	352	96.9	4	1.1	7	1.9
If I did not have diabetes, I think I would be quite a different person?	258	71.1	89	24.5	16	4.4
I do not like being referred to as ‘a diabetic’?	186	51.2	172	47.4	5	1.4
Diabetes is the worst thing that has ever happened to me?	267	73.6	93	25.6	3	0.83
Did you find it difficult to adjust to having diabetes?	290	79.9	71	19.6	2	0.6
There is little hope of living a normal life with diabetes?	175	48.2	159	43.8	29	8.0
Regular exercise keeps diabetes control?	334	92.0	6	1.7	23	6.3
Regular diabetes medication keeps diabetes control?	346	95.6	4	1.1	12	3.3

The majority, 82% of the participants, agreed that ensuring that blood sugar is kept normal would help prevent complications of diabetes. Moreover, a vast majority of 352 (97.0%) of the participants believed that diabetes is a serious disease, and 258 (71.1%) responded that they would have been different persons if they had not had diabetes.

Most participants, 290 (79.9%), said adjusting was difficult when first diagnosed with diabetes. However, just over half of the participants, 51.2%, say they do not like being referred to as people with diabetes. In contrast, in almost similar numbers, 47.4% had no issues being referred to as people with diabetes.

There was almost a balance of views between those participants (48.2%) who believed that there is little hope of living a normal life with diabetes and those participants (43.8%) who thought the opposite. There were 334 (92.0%) participants who agreed that regular exercise helps to keep diabetes in control, while 346 (95.6%) believed that regular diabetes medications keep it controlled.

### Practices related to type 2 diabetes mellitus

Table 3 represents the number of participants who did not have a glucometer machine to help them monitor their blood glucose at home.

**TABLE 3:** Participant’s practices related to type 2 diabetes mellitus.

Questions	Yes		No	
	*n*	%	*n*	%
**Do you have a glucometer machine to test your sugar levels at home?**	158	43.5	205	56.5
**Do you get your blood sugar checked?**	213	56.7	150	41.3
*If yes, how often?*			-	-
Daily	63	29.4	-	-
After a day to a week	50	23.5	-	-
Two weeks to 1 month	55	25.8	-	-
More than a month	45	21.1	-	-
**Do you take diabetes medicine regularly?**	351	96.7	12	3.3
**Do you exercise?**	119	32.8	244	67.2
**Are you adhering to eating low fat plus no-sugar diet?**	269	74.1	94	25.9
**Are you monitoring your body weight?**	137	37.7	226	62.3
*If yes, how often do you exercise?*				
1/2 h per day or 2 h 1/2 per week	46	38.3		
< 1/2 h per day or < 2 h 1/2 per week	11	9.2		
> 1/2 h per day or > 2 h 1/2 per week	16	13.3		
> 30 min per day or > 2 h 1/2 per week	2	1.67		
Occasionally	45	37.5		
**Do you eat vegetables?**	357	98.4	6	1.7
*If yes, how often?*				
Daily	174	48.7		
Weekly	159	44.5		
Twice monthly	18	5.0		
Monthly	6	1.7		

More than half (56.5%) of the participants did not have a glucometer machine at home for monitoring their blood glucose, while almost half (41.3%) said they did not check their blood sugar. Sixty-three participants (29.4%) checked their blood glucose daily, whereas almost a quarter (24.7%) of the participants checked their glucose weekly. Just over a quarter of the participants check their glucose between 2 weeks and a month, while 20.4% only check their blood glucose after over a month.

Almost all the participants (96.7) said they take their medications daily. However, the majority of the participants (67.2%) do not exercise, and most of the participants (62.3) do not monitor their weight regularly. Only 32.8% of the participants engaged in exercise. Of those who exercised, 38.4% exercised half an hour daily, while 37.5% exercised occasionally. The rest of the participants who do some exercise either do less than half an hour per day or less than two and a half hours a week or more than half an hour per day or more than two and a half hours a week.

Most participants (74.0%) adhere to eating low fat and no-sugar diet, while almost all the participants (98.4%) eat vegetables. Nearly half, 48.7%, of the participants eat vegetables daily, while 44.5% eat vegetables weekly.

Table 4 reveals that most participants have good knowledge and attitude with an average practice score towards diabetes.

**TABLE 4:** Participants’ median scores for knowledge, attitudes and practices.

Variable	Good		Average		Poor		Median score of the variable
	*N*	%	*n*	%	*n*	%	
Knowledge scores	245	67.5	89	24.5	29	8.0	16 (out of 20)
Attitudes scores	234	64.5	119	32.8	10	2.8	7 (out of 9)
Practices scores	130	35.8	195	53.7	38	10.5	6 (out of 10)

While the majority of participants (64.5%) showed good attitudes towards diabetes, almost one-third had an average level of attitudes towards diabetes. Despite the participants showing good knowledge and attitudes, only 35.8% had good practices towards diabetes. However, more than half (53.7%) of the participants had an average level of practice towards diabetes, while 10.5% showed poor practice towards diabetes.

## Discussion

This study investigated the level of KAP of diabetes among patients with T2DM in three PHC clinics at the city of Kimberley. This study found that female participants were almost double that of male participants. These findings are similar to a KAP study by Le Roux et al. (females 76.1%, males 23.1%) and Owolabi et al. (females 81.7%, males 18.3%) in the Free State and Eastern Cape of South Africa, respectively.^4,9^ In contrast, Teages Adgoy et al. found an almost equal female-to-male ratio in their study participants,^22^ while Kant and Thapliyal, in a KAP study among T2DM patients in India, reported that males and females accounted for 64% and 36% of participants, respectively.^2^ The difference in findings cited by various authors and our findings may be due to disparities in healthcare-seeking behaviours between males and females in different parts of the world.

The ethnic distribution among patients with T2DM in this study was almost similar to the national population distribution regarding the various ethnic groups in Kimberley. This study found that many participants were black people, followed by mixed-race ethnic groups, then white people and other ethnic groups accounted for the rest.^21^

This study revealed that almost half of the participants had up to grade 12 level of education, while 14.9% of the participants had up to tertiary level of education. Similar studies from other parts of South Africa and India reported low literacy levels among participants.^2,4,18^ This level of education among participants could be attributed to the fact that this study’s location (Kimberley City) is an urban area populated mainly by people working in the central business district.

### Knowledge

This study found that most participants had a good level of knowledge. These findings contrast with similar studies in other parts of South Africa and globally, where they reported poor knowledge of T2DM among participants.^4,9,23^ These disparities in findings may result from this study being done in an urban setting where many participants are likely to be educated and may have access to media, health educational facilities and additional support from allied health workers such as dieticians.^24^

Most participants in this study believed that diabetes medications should be taken for life. This is similar to findings by Le Roux et al., who reported that the majority (85.9%) of participants in their study agreed that diabetes medications are lifelong treatments.^4^ However, this finding did not translate into good practices among the participants in our study. The views regarding the above finding are that all components of KAP and motivation are essential to ensure optimal diabetes control.^25^

### Attitudes

This study also found that most participants had good attitudes towards diabetes. Many participants believed controlling blood glucose, regular exercise and adherence to diabetes medications would help them prevent diabetes complications. This is corroborated by findings in other studies where participants reported good attitudes towards diabetes.^3^ This study found that most participants believed diabetes is a serious disease. Over half of the participants said they dislike being referred to as ‘people with diabetes’. In contrast, Le Roux et al., in their study, stated that over 70% of participants would have no problem being referred to as people with diabetes.^4^

Many participants agreed that people with diabetes should exercise regularly, but only a handful do in practice. This was comparable to findings by Salleh et al., where 99.1% agreed that exercise is essential for people with diabetes, but only a few engage in physical activities.^25^ Also, Le Roux et al. found that only a few participants are involved in regular exercise.^4^

### Practices

Just over one-third of participants had good practices towards T2DM. This was similar to a study by Shawahna et al., where only 36.4% of the participants had good practices towards diabetes.^26^ Another study by Waris et al. likewise reported a low level of practice towards diabetes among participants. They indicated that ensuring people adhered to dietary plans and exercised regularly might be challenging.^27^ In contrast, findings reported by Asmelash et al. in Ethiopia stated that 74.4% of the participants had good practices towards diabetes control.^3^

The participants’ average level of practice towards diabetes control was strongly reflected in their compliance with diabetes diets and exercise. This was supported by Hui Ng et al., who reported that having good knowledge and attitude towards diabetes does not guarantee good practices and management of the disease; instead, a combination of other factors plays a significant role in ensuring better practice among people with diabetes.^28^ The findings in this study reinforce the researcher’s undocumented findings that many of the patients presenting to RMSH Kimberley have diabetes complications. Although they attend the local PHC and may have received good health education, they did not practise it.

This study also found that most participants do not monitor their weights. Weight loss is important in controlling and preventing T2DM.^25^

Many authors have reported a relationship between the level of education in every society and the citizen’s understanding and knowledge of disease patterns. Education provides a platform for a better understanding of our health.^9,23,26,29^ Our study found a significant association between the participant’s level of education and knowledge of T2DM (*p* = 0.0002) and practices (*p* = 0.0075) but not with attitude (*p* = 0.2416) (see Table 5). This finding was supported by Mousavi et al., who stipulated in their study that there is a strong relationship between the participant’s level of education and KAP.^30^ Another study reviewed reported an association between participants’ level of education and attitude towards diabetes but not with practices.^27^ However, Owolabi et al. found no association between education and diabetes knowledge in their study.^9^

**TABLE 5:** Association between the participant’s level of education and knowledge, attitudes and practices.

Variable	Knowledge (*p* = 0.0002)						Attitudes (*p* = 0.2416)						Practices (*p* = 0.0075)					
	Good		Average		Poor		Good		Average		Poor		Good		Average		Poor	
	*n*	%	*n*	%	*n*	%	*n*	%	*n*	%	*n*	%	*n*	%	*n*	%	*n*	%
No formal education	22	9.0	22	24.7	7	24.1	28	12.0	19	16.0	4	40.0	13	10.0	25	12.8	13	34.2
Primary	47	19.2	25	28.1	8	27.6	52	22.2	26	21.9	2	20.0	31	23.9	43	22.1	6	15.8
Up to grade 12	131	53.5	34	38.2	13	44.8	119	50.9	55	46.2	4	40.0	61	46.9	102	52.3	15	39.5
Tertiary	45	18.4	8	9.0	1	3.5	35	15.0	19	16.0	0	0.00	25	19.2	25	12.8	4	10.5

KAP, knowledge, attitudes and practices (*p*-value of < 0.05 is regarded as significant).

This study found a significant association between participants’ knowledge of T2DM and their attitudes and practices towards diabetes, with a *p*-value of < 0.001 and *p*-value of < 0.001, respectively (see Table 6). This was comparable to findings by Alaofè et al., who reported a significant association between good KAP among diabetic patients from four health centres in Benin Republic.^31^

**TABLE 6:** Association between knowledge of type 2 diabetes mellitus and the attitudes and practices among type 2 diabetes mellitus participants.

Knowledge	Attitudes (*p* < 0.001)							Practices (*p* < 0.001)						
	Good		Average		Poor		Total	Good		Average		Poor		Total
	*n*	%	*n*	%	*n*	%		*n*	%	*n*	%	*n*	%	
Good	179	76.5	64	53.8	2	50	245	107	82.3	124	63.6	14	36.8	245
Average	46	19.7	38	31.9	5	50	89	20	15.4	52	26.7	17	44.7	89
Poor	9	3.85	17	14.3	3	30	29	3	2.31	19	9.7	7	18.4	29
Total	234	-	119	-	10	-	363	130	-	195	-	38	-	363

The *p*-value of < 0.05 shows a significant association between the variables.

This study reiterates the findings from other studies in South Africa and globally, where there is an overwhelming conclusion that good KAP is an integral part of successfully controlling and preventing diabetes complications.^9^

Enormous resources are devoted annually worldwide and in South Africa for treating diabetes. Genuine efforts by all relevant role players, such as nurses, dieticians, clinic managers, doctors, family physicians and district health authorities, to ensure good KAP will help reduce the complications of diabetes.

## Strength and limitations

To the best of our knowledge, this study is the first to investigate the KAP of diabetes among T2DM patients attending primary healthcare clinics in the Northern Cape. It could serve as a template for further studies of diabetes and its complications in this province.

This study was limited by the COVID-19 pandemic, which made data collection difficult as some patients did not want to come to the PHC to avoid contracting COVID-19 infection. This study was done in a predominantly urban area with likely a more educated population than most PHCs in South Africa. This may make it difficult to generalise the findings of the study. Convenient sampling was used for the study. This may have been a source of bias because only available patients were selected for the study and may not have accurately represented the population. Another source of bias could be because this study was conducted on all type 2 diabetic patients without considering their comorbidities, their diabetic control or complication status during the data collection period.

Another limitation of this study is that patients with health insurance coverage using private medical centres were not part of this study. This may be a source of bias because most patients with medical aid will likely be employed and more educated.

## Conclusion

This study found that the participants had good knowledge and attitudes towards diabetes but did not translate this into good practices needed to ensure better control and prevent diabetes complications. Educational level was significantly associated with knowledge of type 2 diabetes. The efforts of all stakeholders should be geared towards ensuring that not only do patients with diabetes get enough knowledge about the disease, but they should also have regular counselling and behavioural therapy to link their knowledge with reasonable diabetes control and prevention practices.

## Recommendations

A considerable quantity of resources is spent on diabetes and its complications annually. Healthcare authorities should actively ensure that patients at the PHC level have good knowledge, attitudes and good practice by strengthening the existing healthcare educational resources on diabetes care. Regular education and reinforcement of the importance of ensuring good practice towards diabetes must be emphasised for people with diabetes. Provisions should be made for all people with diabetes to have glucometer machines and diabetes kits, such as glucometer, glucose strips, glucometer battery and instruction manual, to enable them to monitor their blood glucose at home.

Healthcare workers at PHC centres should develop a short tool to evaluate patients’ practices towards diabetes during regular clinic visits.
