# Corrigendum: Diabetes Knowledge, Attitudes, and Practices in adults with type 2 diabetes at primary health care clinics in Kimberley South Africa

**DOI:** 10.4102/safp.v66i1.5922

**Published:** 2024-03-13

**Authors:** Moses Alenbalu, Chika K. Egenasi, Wilhelm J. Steinberg, Omololu Aluko

**Affiliations:** 1 Department of Family Medicine, Faculty of Health Sciences, University of the Free State, Bloemfontein, South Africa; 2 Department of Biostatistics, Faculty of Health Sciences, University of the Free State, Bloemfontein, South Africa

In the published article, Alenbalu M, Egenasi CK, Steinberg WJ, Aluko O. Diabetes Knowledge, Attitudes, and Practices in adults with type 2 diabetes at primary health care clinics in Kimberley South Africa. S Afr Fam Pract. 2024;66(1), a5838. https://doi.org/10.4102/safp.v66i1.5838, there was an error in fourth author’s affiliation.

Instead of:

Department of Biostatistics, Faculty of Family Medicine, University of the Free State, Bloemfontein, South Africa

It should be:

Department of Biostatistics, Faculty of Health Sciences, University of the Free State, Bloemfontein, South Africa

The authors apologise for this error. The correction does not change the significance of study’s findings or overall interpretation of the its results or the scientific conclusions of the article in any way.

